# Bacillus Calmette–Guérin in Immuno-Regulation of Alzheimer’s Disease

**DOI:** 10.3389/fnagi.2022.861956

**Published:** 2022-06-27

**Authors:** Benjamin Y. Klein, Charles L. Greenblatt, Ofer N. Gofrit, Hervé Bercovier

**Affiliations:** ^1^Department of Microbiology and Molecular Genetics, The Hebrew University of Jerusalem, Jerusalem, Israel; ^2^Department of Urology, Hadassah Medical Center, The Hebrew University of Jerusalem, Jerusalem, Israel

**Keywords:** BCG, vaccination, intravesical, Alzheimer’s disease, immunometabolism

## Abstract

Bacillus Calmette–Guérin is frequently the treatment of choice of superficial bladder cancer. Exposing the urinary bladder of elderly patients with bladder cancer to the BCG vaccine reduced the risk of Alzheimer’s disease (AD) substantially. Vaccines against other infectious microorganisms by other vaccination methods showed a similar but a lesser effect. This suggests that immune effects on AD are antigenically non-specific, likely being a metabolic result of immune system activation, similar to that shown for Juvenile diabetes. In this mini review we point to the benefit of BCG vaccine. We then briefly highlight the pathological involvement of the immune system in the AD both, in the peripheral and the central (brain) compartments. Given the uncertain prophylactic mechanism of the BCG effect against AD we propose to take advantage of the therapeutically planned bladder exposure to BCG. Based on pathological aggregation of wrongly cleaved amyloid precursor protein (APP) resistant to the unfolded protein response (UPR) which results in amyloid beta plaques we predict that BCG may impact the UPR signaling cascade. In addition pathways of innate immunity training concerned with energy metabolism, predict capability of activated immune cells to substitute deranged astrocytes that fail to support neuronal energy metabolism. This mini review points to ways through which immune cells can mediate between BCG vaccination and AD to support the wellness of the central nervous system.

## Anticipating Bacillus Calmette–Guérin Prevention of Alzheimer’s Disease

Accumulating data argue for the critical role of the immune system in the course of Alzheimer’s disease (AD). We present here, the potential value of immunotherapy for AD. For the last 30 years immunomodulation of Bacillus Calmette–Guérin (BCG) and its derivatives (EFD-BCG) had been used in animal models of asthma, irritable bowel disease (IBD), multiple sclerosis (MS), and atherosclerosis (Lagranderie and Guyonvarc’h, 2014). In 2018 Faustman’s group at Harvard; found that treatment of the aforementioned animal models can be translated into the use of BCG in humans as well (Kuhtreiber et al., 2018). They reported results of vaccination with BCG of Type 1 diabetic patients, followed for 3 years. The glucose values of vaccinated patients reached glycemic levels close to normoglycemia. Correction of insulin-dependent diabetes by vaccination with BCG seemed a remarkable achievement but may seem enigmatic to most readers. Subsequently a report of Ristori in Rome, stated that BCG had an ameliorating effect in multiple sclerosis (Ristori et al., 2014). Then a challenge from Leslie Norins, a former CDC scientist raised the issue of whether Alzheimer’s disease (AD) could have an infectious agent as its trigger. Oskar Fischer isolated 110 years ago from AD brain autopsies, tubercular-like *Actinobacteria* of the genus to which tubercle bacillus belongs. This sparked the initiative to prevent AD by BCG-vaccination that proved to be a right idea but for a wrong rationale. A retrospective analysis of 1,371 patients treated with BCG instillation in the bladder for superficial cancer, or controls treated by other methods indicated that. BCG reduced the risk for AD by four fold relative to controls (Gofrit et al., 2019a).

## Bacillus Calmette–Guérin’s Non-Specific Effects

Abby and Benn have analyzed clinical results of vaccinations and based on their observations they have coined the term “non-specific effects” (NSE) of vaccines (Benn et al., 2013; Aaby and Benn, 2019). They developed this concept because of the effect of childhood vaccines on off-target illnesses. For example, BCG reduces infant mortality beyond the salvage from TB by some 50%. Calmette, The “C” of the BCG acronym recognized this as early as 1930 (Calmette, 1931) in France where the general mortality in children aged from 1 day to 1 year, as calculated by the dispensaries, varied from 16 to 25% in different departments. In vaccinated children of the same age, living under the same conditions, and controlled by the same dispensaries, tuberculous mortality became nearly zero, and general mortality was four times less than it is in non-vaccinated children. And this striking fact has been observed in every country and in every city where vaccination has been introduced on a large scale for a certain number of years. Calmette queried, “Why should one refuse BCG beneficial influence to children?” and here we ask “and why not vaccinate the elderly?”

Recently a conference was held in Lille France to celebrate the 100th anniversary of BCG. This attenuated bacterium has effects in human disease and conditions way beyond tuberculosis. At the meeting special note was on allergies, and inflammatory and autoimmune diseases. BCG not only appeared as its original self, but as a recombinant organism ready to do more and do it better (see below).

## Focusing on Bacillus Calmette–Guérin and Its Effect on Alzheimer’s Disease

Keeping in mind that BCG might have beneficial effects on AD as it had on type 1 diabetes (T1D) and MS, we examined the rates of AD in countries with or without BCG coverage (Gofrit et al., 2019b). There was a general inverse correlation of low levels of AD risk with high levels of BCG coverage.

## The Approach to Test Bacillus Calmette–Guérin for Alzheimer’s Disease Prevention

With this encouragement, we turned to a situation where BCG is given in large doses and repeatedly, to patients with superficial non-invasive bladder cancer (NMIBC) (Gofrit et al., 2019b). Other than the aggressive BCG treatment, this mode offered the further rationale; the bladder cancer patients are diagnosed at a median age of 73 years (Guancial et al., 2015), most of whom survive more than 5 years after diagnosis, BCG instillation also induces a systemic immune response, and an expected AD prevalence is 4.5% at age 70 and increases each year (up to 15–20% at age 84), so there will be sufficient numbers for statistical analysis. Furthermore, there is the possibility of noting whether there is a BCG dose response.

In a large medical organization data base study of bladder cancer patients (Klinger et al., 2021), the older population (over 75 years, 1578 BCG treated, and 5,147 controls) showed a hazard ratio (HR) of 0.726 (95% CI: 0.529–0.996; *p*-value = 0.0473) for AD. More strikingly, in a hospital-based cohort, BCG treatment resulted in an HR of 0.416 (95% CI: 0.203–0.853; *p*-value = 0.017), indicating a 58% lower risk of developing AD (Gofrit et al., 2019b). The risk of developing PD showed the same trend with a 28% reduction in BCG treated patients, while no BCG beneficial effect was observed for other age-related events such as Type 2 diabetes (T2D) and stroke (Klinger et al., 2021). Finally three studies showed a dose response to the BCG protective effect.

## Independent Confirmation of the Bacillus Calmette–Guérin Effect on Alzheimer’s Disease

Two independent studies in the United States showed a similar picture. Kim et al. (2021) in a retrospective study of a cohort of 1290 NMIBC racially/ethnically diverse patients, 25% of whom were given BCG, found a 60% reduction of risk of AD and other dementia (Guancial et al., 2015). In a study of 26,584 patients with high-risk NMIBC, Makrakis et al. (2021) found that patients with any exposure to BCG (13,477) had a significant 27% lower risk for Alzheimer’s disease compared with no exposure after adjusting for age, sex, race, T-stage, and Charlson Comorbidity Index (Klinger et al., 2021). The authors found a dose response to the BCG with a 46% reduction in patients receiving 12 doses or more. A third study, in this case a prospective one is being conducted at Harvard, which in part is based on these bladder cancer studies (Arnold S, personal communication).

## The Extension of These Studies

Since the hallmarks of AD are brain pathology and cognitive loss, new prospective studies are aimed at producing evidence that BCG affects the pathology detected through biomarkers such as beta amyloid/TAU aggregates and cognition testing. Our own MoCA (Montreal Cognitive Assessment Test for Dementia) study is being coordinated with the Simoa Quanterix system which measures hyper-phosphorylated TAU protein. Another study is applying the mass spectrometry-based plasma amyloid 42/40 ratio along with ApoE (apolipoprotein E) genotypes to produce an Amyloid Probability Score (APS) following BCG administration (personal communication, CT Dow).

## Bacillus Calmette–Guérin Correcting Excessive Hygiene Effects

That BCG can modulate inflammation, whether chronic or acute is a blessing, but why this bacteria and not others? First of all, there may be others, a prime example being a relative, *Mycobacterium vaccae*. Although it has not left its mark on the prevention of TB, it has a record in prevention of leprosy, and even of more interest, as a modifier of stress in mouse models (Reber et al., 2016). Both BCG and *M. vaccae*, Rook has named as “Old Friends” along with other life-long infections which in earlier pre-sanitary eras, he argues, have fine-tuned our immune systems (Rook, 2010, 2018). With modern sterile life he maintains this fine tuning is lost and conditions such as asthma, IBD, ectopic allergies become facts of life. He bolsters his argument by citing the Karelians who have migrated from farm life in Russia to sterile Finland and in the latter “have a fourfold higher prevalence of childhood atopy and a sixfold higher prevalence of T1D than the Karelians on the Russian side of the border”. He redefines the old “hygiene hypothesis” as a “loss of biodiversity.” In this framework BCG is an “Old Friend” which compensates for this “loss.” Without “Old Friends,” pathogens such as herpes and *Porphyromonas gingivalis* can become risk factors of AD (Singhrao and Olsen, 2018; Itzhaki, 2021). Interestingly BCG has been reported to neutralize both the herpes virus and the periodontal bacterium (Hippmann et al., 1992; Kato and Mikami, 2011).

## Molecular Mechanism

Macroscopic pathology in AD shows loss of brain tissue and microscopically extracellular deposition of beta amyloid aggregates accompanied by intracellular hyper-phosphorylated TAU protein aggregates (Veitch et al., 2019). These had been extensively studied for decades and pharmacologically targeted without practical therapeutic result, leading to a reduced effort in drug-discovery for AD by major pharmaceutical companies (Jobke et al., 2018). A change in research direction is badly needed. The recent controversy surrounding Biogen’s Aduhelm has not helped the lack of pharmacological means situation.

## Bacillus Calmette–Guérin Effect on the Inflammatory Processes

### Bacillus Calmette–Guérin Effect on Bladder Cancer

This immunotherapy for non-invasive bladder cancer (Gofrit et al., 2019b) evolved from Pearl’s observation from autopsies that tuberculosis victims suffered less than normal cancer rates (Pearl, 1929). This observation was confirmed by animal studies and was finally applied by Morales et al. (1976) to bladder cancer. The mode of action is not totally clear, but according to Fuge et al. (2015): *“infection of urothelial and bladder tumor cells by BCG results in internalization of BCG, which increases the expression of antigen-presenting molecules. This induces an immune response via cytokine release. Th1 cytokines (IL-2, tumor necrosis factor, IL-12, and IFN*-γ*) and Th2 cytokines (IL-4, IL-5, IL-6, and IL-10) along with IL-8 and IL-17 are all implicated. This complex immune cascade induces antitumor activity mediated by cytotoxic T lymphocytes, natural killer cells, neutrophils, and macrophages.”*

### Regulatory T Cells Immunoregulation of Inflammation

Bacillus Calmette–Guérin and its derivatives could, quoting Lippens et al. (2018) “Mechanistically… impacts the phenotype of plasmacytoid dendritic cells (pDCs), and promotes their ability to induce suppressive IL-10 secreting regulatory T cells (Treg) that inhibit encephalitogenic CD4^+^T cells.” This was further confirmed by the group of Faustman (Keefe et al., 2021) whose findings suggest that BCG has a systemic impact on the T cells of the adaptive immune system, and restores immune balance through Treg induction (Keefe et al., 2021).

#### Innate Immune Training by Bacillus Calmette–Guérin

Monocytes in culture stimulated with BCG as a priming event respond by cytokine secretion such as IL-1β. Upon a second stimulus IL-1β secretion increases (Arts et al., 2018). This response to training by BCG is associated with epigenetic rearrangement of nuclear chromatin (Warburg, 1956; Cheng et al., 2014; Arts et al., 2016a,b,c 2018). With action via Toll like receptors (LTRs), a switch of anaerobic to aerobic glycolysis takes place similar to the Warburg effect in tumors (Warburg, 1956; Semenza, 2010).

#### T Cell Metabolic Switch

Immune training reflects an emerging appreciation of immunometabolism that encompasses innate and adaptive immune cells responding to their respective stimuli (Mathis and Shoelson, 2011). A metabolic switch in T cells occurs upon antigen receptor activation of naïve T cells, switching from low glycolysis/high oxidative phosphorylation (OXPHOS), to aerobic glycolysis, i.e., high glycolysis/low OXPHOS (Finlay, 2013). The switch is associated with increased expression of the glucose transporter and rate limiting glycolytic enzymes resulting in an increased rate of ATP synthesis. At this point, glucose derived carbons are utilized for amino acid and nucleotide synthesis for growth and expansion of T cells supported by the cMyc transcription factor. Most of the pyruvate glycolytic end product is converted to lactate secreted by activated T-cells. Independent of cMyc, IL-2 maintains this metabolic switch through mTORC1 and HIF1 (hypoxia inducible factor 1) complex (Kuhtreiber et al., 2019). IL-2 also promotes T-cell migration to chemokines expressed in inflamed tissues by increasing expression of the chemokine receptor (CCR7) via Akt that enables nuclear activity of FOXO (forkhead transcription reppressors).

#### Bacillus Calmette–Guérin Vaccination Effect on Type 1 Diabetes

Bacillus Calmette–Guérin vaccination of T1D patients resulted in discontinuation of insulin treatment in some of them due to improvement of their blood glucose levels and hemoglobin glycation, although inherent insulin levels were negative. An animal model of T1D showed a similar trend under BCG treatment (Kuhtreiber et al., 2018). Metabolomics’ analysis revealed that BCG treated individual patients and the animals underwent a metabolic switch in immune cells to aerobic glycolysis. Significantly, the T1D was “cured” only after a prolonged period of time post vaccination (3–4 years in humans and 300 days in the mouse model (Kuhtreiber et al., 2018). This raises the question as to how BCG interacts with immune cells along the time span from vaccination to the effective reduction of blood glucose levels. Importantly, what mechanism establishes the sustainability of the non-specific immune sustainability of the BCG effect.

#### Genes in Bacillus Calmette-Guerin That Are Likely to Contribute to Glucose Control

We have identified BCG genes which differ from those of The H37RV strain of *Mycobacterium tuberculosis* (Mtb) based on two dimensional isoelectric focusing and molecular size proteomic analysis (Jungblut et al., 1999). We ruled out proteins expressed by Mtb genes but not by BCG as possible actors which Mtb plays in its association with an increased risk for diabetes (Yorke et al., 2017). There were, however, genes expressed by BCG, but not by the Mtb genome (Jungblut et al., 1999). These are more likely candidates for controlling glucose in T1D. For this comparison NCBI accession numbers in parallel to the Rv numbers of Mtb were used to mirror and represent the BCG proteins instead of the different nomenclature of BCG. In Table 1 selected gene products from the BCG slab gel were compared with respective gene products from the parallel Mtb bacilli gel, based on density of the protein spots (Jungblut et al., 1999).

**TABLE 1:** Bacillus Calmette–Guérin proteins overexpressed vs. mycobacterium Mtb proteins.

No.	BCG (Chicago)	TB (H37Rv)	NCBI accession no.	Protein	Rv name	Function
1	Up	Down	585892	L7/L12 50S ribosomal protein	Rv0652	Activates inflammatory pathway (TLR4, TNFa, IL-6 IL-1b) IFNg (Lee et al., 2014)
2	Up	Down	2127455	AhpC/TSA family	Rv2429	against oxidant and nitrozant reaction of macrophages (Alharbi et al., 2019)
3	Up	Mobility variant	1452900	*S*-adenosyl-methionine syntase	Rv1392	Produces the major methyl donor (Chiang et al., 1996)
4	Up	None	1817673	G6PD	Rv0407	Possible host switch to aerobic glycolysis (Stincone et al., 2015)
5	Up	None	2072672	SimilarProtects to Soj protein	Rv3213c	Mycobacterial proteasomal substrate, chromosome partitioning (Hu et al., 2018)
6	Up	Down	2104386	Neuraminidase?	Rv3463	Enhances bacterial phagolysosomal fusion in host (Park et al., 2019)
7	Up	Down	1781160	Contains pyridoxal phosphate attachment site	Rv3054c	Aminotransferase class II
8	Up	None	2143298	Arylsulphatase	Rv0663	Intermediate and lipid metabolism and respiration
9	Up	Mobility variant	1694860	Oxidoreductase aldo/keto family	Rv2971	Effector oxidative damage, NADPH cofactor (Picklo et al., 2001)
10	Up	Mobility variant	2808725	Oxidoreductase	Rv0068	Oxidoreductase, respiration intermediate metabolism

Most of these proteins are involved in energy metabolism, the closest to aerobic glycolysis is (No 4, Table 1); G6PD which is overactive in parallel with the increased glycolysis during the metabolic reprogramming in innate immune cells. It is too early to conclude that the prokaryotic G6PD of BCG is functionally supporting/replenishing the eukaryotic G6PD of the hosting macrophages in driving more glucose-6P into the PPP shunt, and thus expediting consumption of excess blood glucose. In addition, an overactive PPP (whether in the BCG bacterium or by the host cell under Warburg effect) should generate more NADPH to neutralize oxidant radicals of macrophages which are harmful to BCG. Thus assisting proteins No. 2, 9, and 10 of BCG (in Table 1) which use NADPH as a cofactor and keep BCG protected from the host. Intense generation of NADPH in the parasite BCG or host macrophage may be a marker for increased blood glucose consumption. *S*-adenosylmethionine (SAM) synthase (protein No. 3 in Table 1) raises the question of a possible assistance to the host eukaryotic host function. SAM is a major methyl group donor being part of the biochemical trans-sulfuration pathway, which in animal cells fulfills three main functions. Firstly, methyl groups are contributing to epigenetic alterations of gene expression (immune training?). Secondly, the same pathway is engaged in nucleotide synthesis. Thirdly, oxidative damage is neutralized via the glutathione which is part of the same pathway (Reed et al., 2008).

Table 1 exhibits protein expression differences between H37Rv Mtb strain and the Chicago BCG strain. However, when the same Mtb strain was compared to the Tokyo172 BCG strain the differences of only proteins 1, 2, 5, 6, and 9 from Table 1 were found. This underscores the variability in attenuation outcome between the many BCG vaccine preparations available worldwide.

#### Energy Metabolism in the Brain, Cooperation Between Neurons and Glia

Metabolic cooperation between neurons and glia has been claimed to occur since the mid1990s and has become a controversial issue (Allaman et al., 2011; Belanger et al., 2011). The metabolic cooperation has been based on a so called “astrocyte-neuronal lactate shuttle” (ANLSH) whereby lactate as the glycolytic end product secreted by astrocytes is transported into neurons via monocarboxylate transporters (Allaman et al., 2011; Belanger et al., 2011). According to proponents of this shuttle, inside the neurons lactate is converted to pyruvate that is utilized by the Krebs cycle in mitochondria to generate ATP in a process that doesn’t require neuronal glucose (Stincone et al., 2015). Yet neurons express a glucose transporter (GLUT3) active in glucose intake and potentially adding to controversy over lack of neuronal glycolysis (Simpson et al., 2007). The apparent glucose intake by neurons is explained by its diversion to the aerobic arm of the pentose phosphate pathway (PPP) (Stincone et al., 2015). The low efficiency of the glycolytic pathway in cortical neurons is explained by the lack of Pfk/fb3 (6-phosphofructo-2 kinase fructose-2 6 bisphosphatase), the most potent enzymatic activator of glycolysis. The lack of Pfk/fb3 is ascribed to APS/C subunit 1 (anaphase promoting complex subunit 1) which is highly active in cortical neurons and shows low activity in astrocytes (Herrero-Mendez et al., 2009). This is consistent with abundant glycolysis (the source of lactate) in astrocytes and lack of glycolysis in cortical neurons. Inversely, this explains the preferential utilization of glucose by the PPP shunt in neurons and the use for glycolysis in astrocytes. The PPP shunt provides synthetic building blocks for cell growth and maintenance and importantly NADPH reducing-equivalents for scavenging free radicals in protection of neuronal wellness. Astrocyte impairment has been blamed as a cause of brain damage in AD (Ding et al., 2013; Gonzalez-Reyes et al., 2017), and it is therefore possible that immune cells such as T-cells and monocytes reaching the brain are neuroprotective rather than neuro destructive (Schwartz, 2017). Neuroprotection by infiltrating activated immune cells can be explained by the energy metabolism contribution of lactate in assistance to astrocyte-neuron cooperation as opposed to classical immunological activity of tissue-antigens targeting.

## Other Vaccines

Although the immense spread of the NSE (non-specific effect) of BCG has long been recognized, this characteristic of adult vaccination did not extend to other vaccines. When an anti-influenza vaccine aimed at the subtype HnNn reduced the incidence of other influenza subtype could reflect the NSE phenomenon although NSEs were probably not in the mind of vaccine providers. Perhaps an early “indication” that vaccines in adults and not only in children have non-specific effects was the study of Verrault in 1989 on vaccines and dementia (Verreault et al., 2001). They found that routine vaccinations reduced the incidence of AD. Since our publication of the BCG prophylactic effect against AD risk (Gofrit et al., 2019b), there has been a cascade of other vaccine studies, purporting to reduce the risk of AD. These now include, anti-influenza, anti-pneumococcal, TDaP, and anti-shingles. The reductions in AD risk have ranged from 17 to 43% (Verreault et al., 2001; Greenblatt et al., 2021; Scherrer et al., 2021a,b).

## Limitations

Our bladder cancer study has been examined for its bias in the exclusion of “frail” individuals, judged not capable of enduring the BCG treatment (Gofrit et al., 2019b). A critique of the results of the other vaccines has been subjected to the bias we have termed, the healthy individual syndrome, “healthy non-smoking, non-drinking people who exercise get vaccinated, while those not health concerned individuals who do all the things you shouldn’t do” become demented. Hopefully well-designed prospective studies will bring clarity to these results.

### Involvement of the Immune System in Alzheimer’s Disease

In the 1990s the involvement of the immune system in AD has been suspected due to several experimental results; (a) the increase of CD8 suppressor/cytotoxic T cells (but less differentially CD4 helper cells) were found in the blood of AD patients (Singh, 1996; Singh and Cheng, 1996), (b) Serum S100 protein was elevated and exhibited *in vitro* binding of the 40 amino acid residues of β-amyloid peptides (Ab), and (c) the C1q complement component was elevated. At that time the functional impact of CD8 T cells from outside the brain on the brain itself was not clarified, whether it acts by suppression of autoimmune cells cytotoxic toward microglia, or by directly killing microglia. Although at that time, the role of these peripheral blood T cells in AD hasn’t been determined, however they have suggested that the immune system is involved in the pathology of AD. Since the discovery (six decades back) of Ab aggregates in brains of AD they are considered the most prominent histopathological biomarker of AD. While traditionally Ab have been considered neurotoxic there was no proof, even *in vitro*, that Ab can directly kill neurons (Marx et al., 1999). The closest evidence for the ability of Ab to influence neurons was their effect on neuronal morphology and function (Iversen et al., 1995). Notably, Interferon-γ (IFNγ) inhibited production of amyloid precursor protein (APP) and secretion of its cleaved oligomers, this IFNγ activity counteracts the stimulation of APP by interleukin-1 (IL-1) inflammatory cytokine (Marx et al., 1999) in thyroid cells (outside the brain!). The non-secretory cleaved oligomers were localized to specific intracellular compartments (Marx et al., 1999), starting at the Golgi apparatus some of them were transferred to lysozymes (Haass and Selkoe, 1993). This is interesting because in the brain IFNγ stimulates astrocytes to express major histocompatibility receptors class I [MHC-I (Jarosinski and Massa, 2002) and MHC-II (Lee et al., 1992)]. It implies that if astrocytes may present on their surface (self) antigenic fragments via MHC-I receptors (presentation is presumably to adaptive immune cells), it is consistent with the nature of presentation of fragmented APP oligopeptides (secretory type) as an immunological self-identification. If IFNγ or its functional opponent IL-1, will inhibit expression and/or processing secretory oligopeptides, this could result in the aberrant 42 peptide pathological Ab uncleavable aggregates. This may render them unprocessable for antigen presentation, and resistant to clearance by quality control chaperons by the unfolded protein response (UPR). The increase in AD of serum S100 calcium binding protein (Singh, 1996), implies that UPR is involved in cellular stress that requires chaperones to clear misfolded peptides in order to restore protein/peptide homeostasis. S100 and inflammatory molecules are also overexpressed in AD brain astrocytes (Akiyama et al., 2000), reflecting immune activities that are taking place concomitantly at both sides of the blood brain barrier (BBB) (Bettcher et al., 2021). S100 interacts with several heat shock protein chaperons (see S100A interactome at the GENECARDS website) that normally may function in intracellular clearance of misfolded peptides. Peripheral immune activities typical in AD individuals are reflected by central (in-brain) immune reactivity that may result from cells crossing the BBB (Bettcher et al., 2021). There are cases of peripheral immune activation, such as peripheral Th2 pathological cell activity, associate with AD without any detected Th2 infiltration in the CNS (Cao et al., 2009), such effects may possibly be mediated across BBB by extracellular vesicles (Monsonego and Weiner, 2003). Adult vaccination against several infections has reduced the risk of developing AD (Greenblatt et al., 2021) antigenic wise non-specific. The most prominent effect was obtained by intravesical BCG treatment of superficial bladder cancer patients (Gofrit et al., 2019b). This implies that the antigenic quality of the non-pathological immune activation by vaccines is less important than the quantitative metabolic consequences of vaccination. Therefore, in our opinion the present experimental priority lays in localizing the differential BCG impacts in steps of signaling pathways concerned with the integrated stress response factor eIF2a. In parallel with these signals, levels of the autophagy initiation biomarker are relevant. Also the cytosolic activation/inhibition status of the inflammation master transcription factor NF-kB is relevant. These pathways, which we are analyzing, work coordinately in some cellular systems (Klein et al., 2013, 2014, 2016, 2017) and may provide insight as to where in these pathways BCG makes the most prominent impact (within peripheral blood mononuclear cells). Ideally such experiments should be done on blood samples of BCG treated patients under similar conditions that resulted in prevention of AD (Gofrit et al., 2019b).

## Summary

1. Although this focused review stresses the “proof of concept” that BCG can reduce the risk of AD, intravesical BCG cannot be entertained as a method of immunotherapy or immunoprophylaxis in normal adults.
2. It does, however, argue that the living BCG organism acts systemically, and its administration by other routes proves this. In Brazil oral BCG continued in use until the late 1980’s (Monteiro-Maia and Pinho, 2014) and even as a further test it was used successfully to treat superficial bladder cancer (Netto Junior et al., 1978).
3. Further proof of the systemic action of BCG, has been provided by Hoft et al. (2018) whose studies of PO and ID BCG have shown that both give a systemic Th1 and IFN-γ response, although PO administration yielded a better IgA and bronchoalveolar Tcell reaction and ID application a better Th1 one.
4. Strains of BCG show variation in their effectiveness, but research aimed at understanding these differences and engineering the bacterium to even greater effectiveness is underway (Angelidou et al., 2020).
5. Lastly, routine vaccinations for elderly adults, whether or not the beneficial effect on dementia is overstated, should be considered on a patient’s check list by every geriatrician.

## Author Contributions

BYK added the metabolic mechanism to BCG action along with the trained immunity. ONG was the surgeon whose clinical application of BCG was central to this study. HB was a renowned BCG expert and he brought to the clinical trial. CLG was a microbiologist who connected the dots between BCG as a vaccine and BCG’s non-specific effects. All authors contributed to the article and approved the submitted version.

## Conflict of Interest

The authors declare that the research was conducted in the absence of any commercial or financial relationships that could be construed as a potential conflict of interest.

## Publisher’s Note

All claims expressed in this article are solely those of the authors and do not necessarily represent those of their affiliated organizations, or those of the publisher, the editors and the reviewers. Any product that may be evaluated in this article, or claim that may be made by its manufacturer, is not guaranteed or endorsed by the publisher.
